# New Automatized Method of 3D Multiculture Viability Analysis Based on Confocal Imagery: Application to Islets and Mesenchymal Stem Cells Co-Encapsulation

**DOI:** 10.3389/fendo.2018.00272

**Published:** 2018-05-25

**Authors:** Clovis Chabert, Camille Laporte, Arnold Fertin, Emily Tubbs, Cécile Cottet-Rousselle, Florence Rivera, Magali Orhant-Prioux, Anaick Moisan, Eric Fontaine, Pierre-Yves Benhamou, Sandrine Lablanche

**Affiliations:** ^1^Grenoble Alps University, Laboratory of Fundamental and Applied Bioenergetics (LBFA), Environmental and System Biology (BEeSy), Grenoble, France; ^2^INSERM U1055, Grenoble, France; ^3^Laboratory «Adaptations au Climat Tropical, Exercice et Santé» (ACTES; EA 3596), French West Indies University, Pointe-à-Pitre, Guadeloupe, France; ^4^CNRS, TIMC-IMAG, University Grenoble Alpes, Grenoble, France; ^5^Microsyst. for Biol. & Health Department, CEA-LETI, Grenoble, France; ^6^Cell Therapy and Engineering Unit, EFS Auvergne Rhône Alpes, Saint Ismier, France; ^7^Grenoble University Hospital, Grenoble, France

**Keywords:** pancreatic islets, transplantation, co-encapsulation, mesenchymal stem cells, automatization, viability analysis

## Abstract

Co-encapsulation of pancreatic islets with mesenchymal stem cells in a three-dimensional biomaterial’s structure is a promising technique to improve transplantation efficacy and to decrease immunosuppressant therapy. Currently, evaluation of graft quality after co-encapsulation is only based on insulin secretion. Viability measurement in a 3D conformation structure involving two different cell types is complex, mainly performed manually, highly time consuming and examiner dependent. Standardization of encapsulated graft viability analysis before transplantation is a key point for the translation of the method from the bench side to clinical practice. In this study, we developed an automated analysis of islet viability based on confocal pictures processing of cells stained with three probes (Hoechst, propidium iodide, and PKH67). When compared with results obtained manually by different examiners, viability results show a high degree of similarity (under 3% of difference) and a tight correlation (*r* = 0.894; *p* < 0.001) between these two techniques. The automated technique offers the advantage of reducing the analysis time by 6 and avoids the examiner’s dependent variability factor. Thus, we developed a new efficient tool to standardize the analysis of islet viability in 3D structure involving several cell types, which is a key element for encapsulated graft analysis in clinical practice.

## Introduction

Islet transplantation is a promising therapy for millions of patients with type 1 diabetes as it offers a perspective of an efficient metabolic control with a prevention of severe hypoglycemia without insulin injections (1–3). However, islets graft success may be compromised by grafted islets exposition to an acute stress due to ischemia reperfusion injuries associated with an instant blood-mediated inflammatory reaction and to chronic rejection (4–9). Moreover, to prevent graft rejection, an immunosuppressive therapy is required, which can lead to severe adverse events such as infection or neoplasic disease.

To avoid immunosuppressant therapy and to maintain graft viability and functionality over time, several approaches are currently studied. Among them, islet encapsulation in a permeable three-dimensional structure seems highly promising (10). The capsule allows the diffusion of insulin, oxygen, and nutriments while protecting islets from the immune system (11). This technique has already shown interesting results in different species (12–14) and paves the way for free-immunosuppressive islets transplant therapy (15). To increase the efficacy of encapsulation, Veriter et al. have shown the benefits of co-encapsulating islets with mesenchymal stem cells (MSCs) (16). Indeed, MSCs are well known for their *in vitro* and *in vivo* protective effects on islets viability (17) and functionality (18) as well as their pro angiogenic effect (19) under different stress conditions (20). Thus, co-encapsulation of islets with MSCs appears to be a promising approach to improve treatment strategies for diabetic patients (21).

However, the translation from the bench side to patients is facing various obstacles (22). The validation of these complex capsules containing two different cell types requires an accurate analysis of the viability and functionality of the co-encapsulated cells. The reference method to evaluate islets viability is the fluorescein diacetate (FDA) and propidium iodide (PI) or ethidium bromide (EB) co-staining (23, 24). This method, usually performed by islet isolation centers to characterize and validate islet preparation before transplantation, can be limited in a complex multiple cell type culture system. Indeed, this double staining does not allow to discriminate the viability of the two different cells types. The other probes used to replace FDA as Calcium AM, SYTO^®^13, SYTO^®^24, and SYBR^®^14 are also limited to a monoculture system when coupled to IP/EB (25–27). These probes can be used for flow cytometry viability analysis, but the dissociation of the capsule is compulsory as well as the dissociation of islets before acquisition. This dissociation enables a precise analysis but could induce a cellular stress which could lead to altered viability analysis. Thus, the current manual counting under microscope of the stained cells is the only tool available to determine the viability of islets included in an unmodified 3D structure such as a capsule. This technique is operator dependant and highly time consuming (28) which is poorly adapted in the emergency context imposed by transplantation.

Due to these limitations, most of the current studies on islets co-encapsulation are mainly based on an evaluation of the insulin secretion functionality (26, 29) restricting analysis of the capsules to functionality analysis and neglecting cells viability analysis. Thus, the development of a new standardized tool to characterize the viability of each cell type in the capsule content is highly suitable and constitutes a key point to further develop this treatment strategy.

The aim of this study was to develop and validate a standardized method to substitute the current manual viability analysis of islets co-encapsulated with MSCs, in agreement with the clinical constraints. For that purpose, we propose an automated picture analysis of stained cells obtained by confocal microscopy.

## Materials and Methods

### Animals

Two adult Wistar male rats (350 g) were used in this study. Procedures were carried out in accordance with European Directives 86/609/EEC, 2010/63/UE well-being and treatment of animals. All the procedures were approved by the ethics committee affiliated to the animal facility of the university (D3842110001) and agreed by the French Ministry of Research (9998_LBFA-U1055).

### Islets Isolation Procedure and Islets Culture

A cannula is inserted in the rat’s Wirsung duct to permit the pancreas perfusion with collagenase type IX, 1 mg/ml (Sigma). Perfused pancreas was removed and digested in warm bath at 37°C for 11 min as previously described (30). Islets were then purified by Histopaque (Sigma-Aldrich, St. Louis, MO, USA) density gradient centrifugation. Islets are seeded in RPMI 1640 supplemented by 10% fetal bovine serum (Sigma-Aldrich, St. Louis, MO, USA), 2 mM l-glutamine, 100 U/ml penicillin, 100 µg/ml streptomycin, and 1 mM sodium pyruvate (Pan Biotech, Aidenbach, Germany). Islets were incubated at 37°C in a humidified atmosphere (95% air–5% CO_2_) and cultured for 12–24 h before encapsulation.

### MSC Culture

Mesenchymal stem cells were isolated from bone marrow aspirate from four healthy donors who gave their written informed consent. All procedures were in compliance with the French public health code (Article L1241-1) and were performed by the Cell Therapy and Engineering Unit of EFS Auvergne Rhone Alpes. After thawing, MSCs are seeded at 3,500 cells/cm^2^. Cells are maintained in culture at 37°C and 5% CO_2_ in MEM alpha media (Sigma-Aldrich, St. Louis, MO, USA) supplemented by 10% fetal bovine serum (Sigma-Aldrich, St. Louis, MO, USA), 2 mM glutamax, 100 U/ml penicillin, and 100 µg/ml streptomycin (Thermo Fisher Scientific, Waltham, MA, USA). Media are changed every 3–4 days. When the cell density reached 80–90%, cells were split with Trypsin–EDTA (0.25%), phenol red (Thermo Fisher Scientific, Waltham, MA, USA).

### Encapsulation

All islets and MSCs are encapsulated in one session at the Laboratoire d’électronique des technologies de l’information of the Commissariat à l’Energie Atomique et aux énergies alternatives (CEA-LETI, Grenoble, France) according to a microfluidic method. The pancreatic islets are washed in a buffer combining 150 mM NaCl, 20 mM HEPES, and 1 µg glucose. The islets are included in a solution of 2.5% alginate, 150 mM NaCl, 20 mM HEPES, 1 g/l glucose, and encapsulated in the microfluidic encapsulation system. All the steps are integrated in a single-use cartridge allowing oil phase capsule formation, gelation of the capsules in a calcium bath, and harvesting in the buffer. The encapsulated islets are returned to culture in complete RPMI 1640 medium.

### Cells Staining

To differentiate the two cell types included in the capsule, MSCs were stained by PKH67 (Sigma-Aldrich, St. Louis, MO, USA) that labeled lipid membrane structure (λ_exc_\λ_em_ = 490/504 nm) before encapsulation. Briefly, as previously described (31) MSCs were dissociated with trypsin 1× and wash with Dulbecco’s phosphate-buffered serum (PAA Laboratories, Velizy Villacoublay, France). Then cells were suspended in diluent C with 2 µM of PKH67 during 5 min. The staining reaction was stopped by adding fetal bovine serum. MSCs were then washed three times with complete media before encapsulation.

Before image acquisition, capsules containing cells were stained with 2.21 × 10^−10^ mol/ml Hoechst 33342 (λ_ex_/λ_em_ = 361/497 nm) during 15 min. Just before pictures’ acquisition, 1.50 × 10^−10^ mol/ml of PI (λ_ex_/λ_em_ = 545/630 nm) (Interchim, Montluçon, France) was added to the medium containing capsules. Hoechst stains nuclei of living cells in blue while PI stains nuclei of necrotic cells in red. MSCs previously stained by PKH67 are labeled in green (32). Thus, co-localization of PI or Hoechst stain along with the PKH67 signal permits to analyze MSCs’ viability whereas nuclei out of the green signal provides data regarding islets cells viability.

### Pictures Acquisition

Images were collected with an LSM710 AxioObserver Airyscan inverted laser scanning confocal microscope (Carl Zeiss Enterprise, Oberkochen, Germany) equipped with a 20× oil dry objective (Plan apochromat 20×/0.8). Laser excitation was 405 nm for Hoechst, 488 nm for PKH67, and 561 nm for PI. Fluorescence emission detected by GaAsP detectors was 410–485 nm for Hoechst, 493–551 nm for PKH67, and 571–640 nm for PI. Confocal pinhole (Airy units) was 1 for all channels. Five z-stacks of 13–22 pictures (total = 77 pictures) with 10 µm z-step of optical sections were acquired and saved in TIFF format (1,024 × 1,024 pixels).

### Manual Viability Analysis

To avoid bias, manual analysis were blinded and performed by four different examiners which did not know the results obtained with the automatized method. Each experimenter merged the green channel (corresponding to the MSC cytosols) with the blue or red channel. Then, selection of nuclei present in or out the green signal was visually performed on each picture of the stacks using the “Multi-point” tool of ImageJ 1.51. ImageJ is an open source software especially developed for picture analysis and widely used in the world (33). The viabilities presented in this article are calculated by dividing the number of nuclei stained with Hoechst by the total number of nuclei (number of nuclei stained with PI + Hoechst).

### Automated Viability Analysis

**Figure 1:**
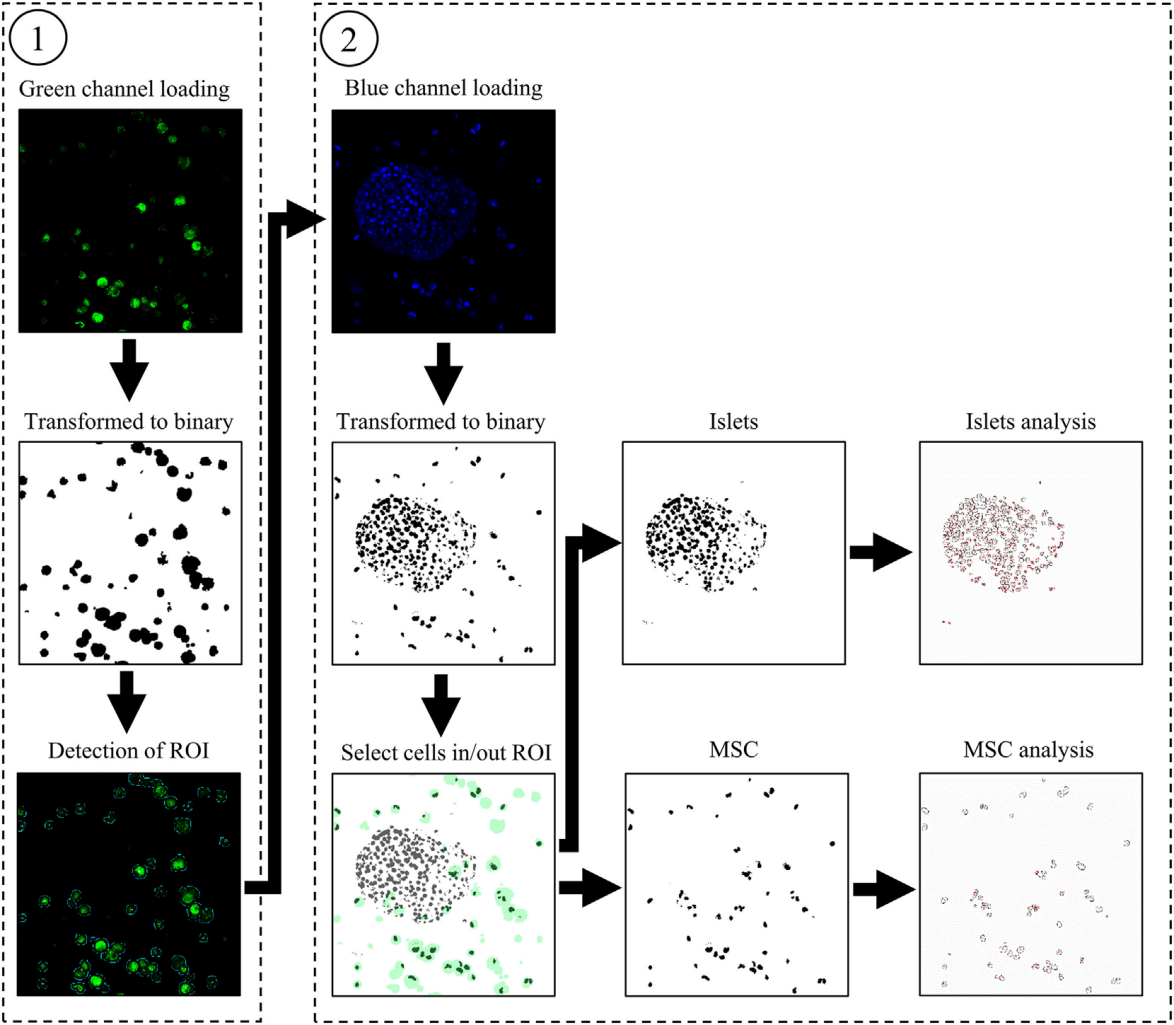
Graphic representation of the automated analyses of the green ① and blue channel ② to detect living nuclei of islets and mesenchymal stem cells.

Regarding our automated method developed, previously acquired pictures were analyzed by a code elaborated with the ImageJ interface. The description of the code can be separated into four main steps as presented in Figure 1.

Once the software is started, a window is displayed to set up several variables. First, fields to fill in allow to entry the folder’s pathway containing the picture files, name, and numeration. This window also permits the configuration of the channel appellation and different filters concerning the minimal and maximal thresholds accepted during the binary transformation, the factor of regions of interest (ROIs) dilatation containing the detected MSCs, and a filter to suppress signal under the determined surface. Two check boxes allow to enhance the blue channel contrast if the signal is too low and to choose or not to analyze the viability of MSCs.

Then, the code loads the green channel, corresponding to the MSCs cytosol staining. The picture is transformed into a binary signal; the detection of the green areas is performed and saved as ROI. All ROIs detected are saved in the folder named “…/Analyse/ROI_Save.” Then, blue channel, corresponding to the living nuclei is loaded and transformed into a binary picture. In each ROI previously saved, the *x, y* coordinates of MSCs’ nuclei are detected and saved in a matrix. The same analysis is performed with the red channel. The next step corresponds to the suppression of the MSCs’ nuclei in blue and the red channel. For that step, we used the “MagicWand” function allowing a selection of all the black dots around the different *x, y* coordinates and replacing them by white dots. To avoid artifacts due to the analysis of the same nucleus on several slides, the code is able to detect if nuclei are present on the next slide at the exact location of those previously detected and if so, to delete the signal. Then, each picture of blue channel is saved in “…/Analyse/Islets_Nucleus” and red channel in “…/Analyse/Necrotic_Islets_Nucleus.” If the option “Analyse MSCs parameters:” is checked at the beginning of the program, the nuclei present in ROIs are analyzed and save in the folder “…/Analyse/MSC_Nucleus” for blue channel, and “…/Analyse/Necrotic_Islets_Nucleus” for the red channel. Finally, the number and area of the islets and MSCs’ nuclei detected in each picture are saved in the folder “…/Analyse.” An algorithmic representation of the software main steps and the entire code are available in open source, downloadable in Supplementary Material.

### Statistical Analysis

All data are presented in Figure 3A as mean ± SD and were analyzed using a one-way ANOVA as they were normally distributed. The correlation coefficient represented in Figure 3B was calculated as a Pearson product moment.

## Results

Figure 1 represents intermediate steps used by the software to analyze the different cell type’s viability. The Figure 1① shows the transformation of the initial green channel picture to a binary signal to allow the detection of the green areas. The last picture of Figure 1① shows the ROIs that are automatically saved by the software during analysis. In Figure 1②, the three mains steps of the blue channel analysis are as follows: the binary conversion, the selection of islets or MSCs’ nuclei, and the analysis are displayed. Once the blue channel is transformed in a binary signal, the picture is merged with the ROIs previously detected. Nuclei of MSCs or islets are automatically selected as black dots and black dots are segmented to precisely count the number of nuclei. Modified pictures used for analysis are those saved by the software.

**Figure 2:**
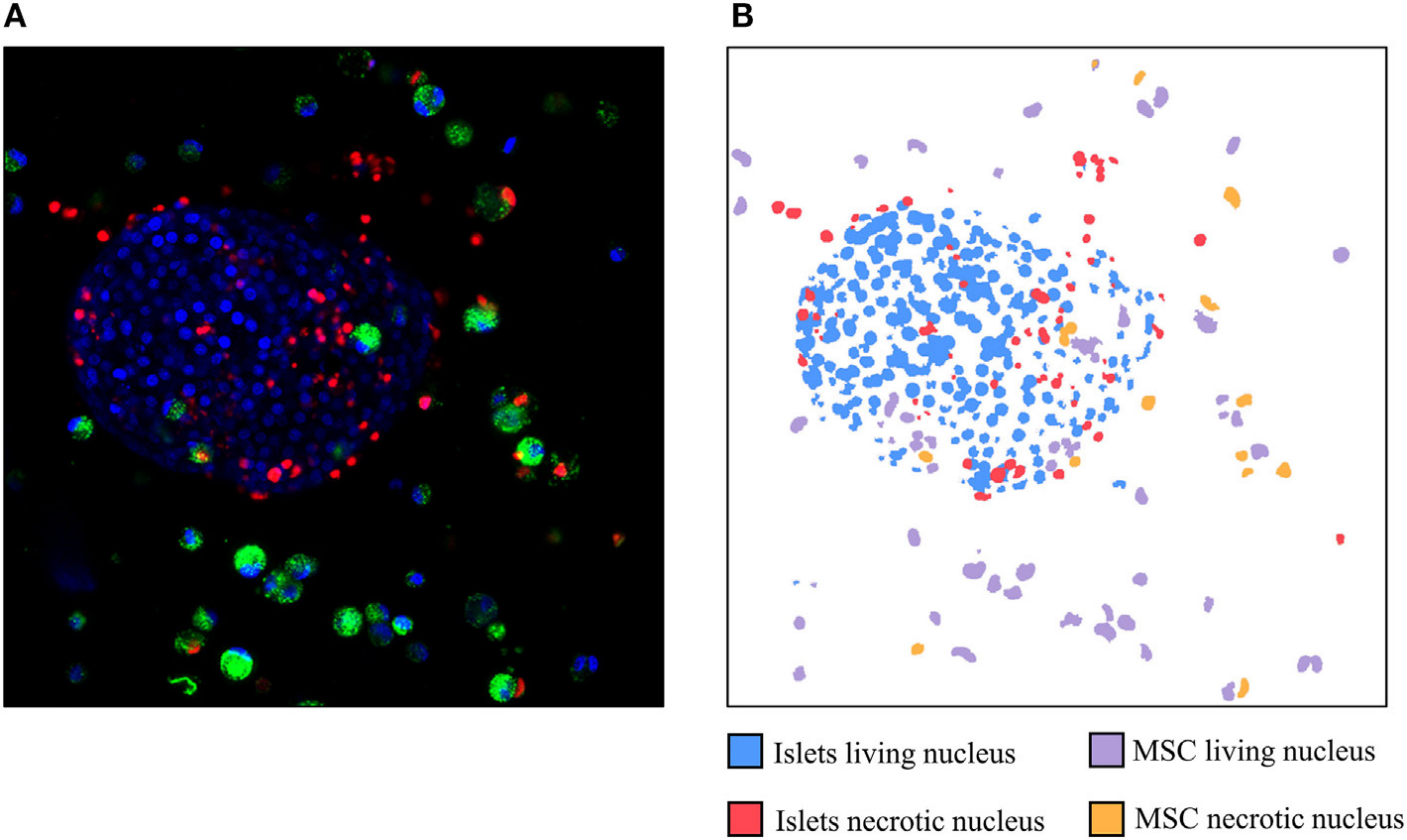
Example of a picture obtained by confocal microscopy **(A)** and the identification of the different nuclei detected by the software **(B)**. Red: necrotic nuclei (IP); green: mesenchymal stem cells cytosol (Pkh); and blue: living nuclei (Hoechst stain).

Figure 2 corresponds to the three channels merged in an initial picture (Figure 2A) and the identification of each nucleus by the software (Figure 2B). Nuclei considered by the software as living islets appear in blue whereas MSCs appear in purple. Islets nuclei detected as necrotic appear in red whereas MSCs are drawn in orange. This figure allows to visually confirm the automated identification performed by the software.

**Figure 3:**
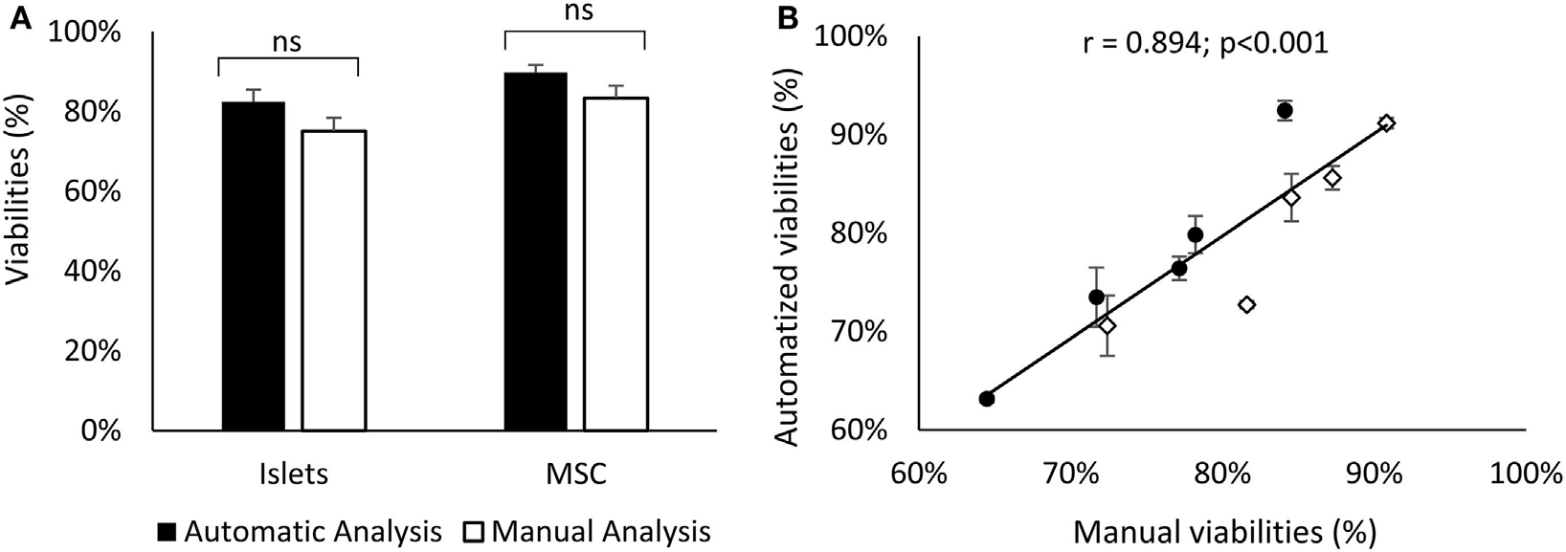
Comparison of the islets (black) and MSCs (white) viability obtained manually or with the software **(A)**. Correlation between these two methods for islets (dots) and MSCs (diamonds) cells **(B)**. Errors bars of panel **(B)** correspond to variability of viabilities manually obtained by different experimenters. Results represent the mean ± SD; *n* = 5. Abbreviation: MSCs: mesenchymal stem cells.

In Figure 3A, the mean viability of islets and MSCs detected manually is compared with the automated software approach using five stacks of pictures containing between 14 and 22 optical sections. This figure shows no difference between the viability of the islets detected manually or detected by the software (76.02 ± 6.3 vs. 77.07 ± 10.6%, respectively, ns). No differences were observed when comparing MSCs’ viability analyzed with the two techniques (manually: 83.20 ± 6.5% vs. software: 80.74 ± 8.8%, ns). The visual resemblance between these two techniques, showed in Figures 2 and 3A, is confirmed by the tight correlation presented in Figure 3B. Indeed, the correlation of the viability manually evaluated with the viability automatically determined by the software for MSCs and islets shows a coefficient of correlation over 0.89 and a *p*-value lower than 0.001.

## Discussion

This study provides evidence of the efficacy and reliability of the automatized techniques we have developed using the ImageJ interface to analysis cells viability in a 3D structure. The high similarity of viability measured by two different methods (less than 3% of variation) and the tight correlation between them (*r* = 0.894; *p* < 0.001) validate the results obtained with our technique. Moreover, the automated analysis tool suppresses the variability of the viability measured by different examiners (more than 5% in this study, see SD in Figure 3B). The standardization of viability analysis is a key point to determine common standardized quality criteria for islet preparation.

The automated method developed in this study allows to strongly reduce the time spent for analysis in comparison with the manual technique. Our results showed a decrease of the analysis duration from approximately 1 h 45 min to 15 min for users trained to the macro’s utilization. The saving process during the analysis steps allows a visual feedback of the software results, and a check of analysis if desired.

The availability of the code in Supplemental Material allows users with programming skills to perform few modifications to adapt the software to many different applications. Thus, this code allows the viability measurement of different cells type in all complex tissues which cannot be mechanically separated.

As co-encapsulation of islets and MSCs appears to be a highly promising technique to major the efficacy of islets graft in the diabetes medical care, we have developed a new standardized approach to measure and distinguish the viability of co-encapsulated cells through an automated and time saving method preserving the 3D conformation of the structure.

## Ethics Statement

This study was carried out in accordance with the recommendations of European Directives 86/609/EEC, 2010/63/UE well-being and treatment of animals. The protocol was approved by the “Comité d’éthique en expérimentation animale de Grenoble” and by the French Ministry of Research (No. 9998_LBFA-U1055).

## Author Contributions

CC and CL participated equally in the design, measurement, analysis, and redaction of this work. AF, CC-R, and MO-P contributed to the data measurement and analysis. FR, AM, EF, and P-YB participated to the design of this work. SL and ET participated to the design and the redaction of this work.

## Conflict of Interest Statement

The authors declare that the research was conducted in the absence of any commercial or financial relationships that could be construed as a potential conflict of interest.
