# Supplementary material for: New Automatized Method of 3D Multiculture Viability Analysis Based on Confocal Imagery: Application to Islets and Mesenchymal Stem Cells Co-Encapsulation
Source: Front Endocrinol (Lausanne). 2018 May 25;9:272. doi: 10.3389/fendo.2018.00272 (PMC5980978; doi:10.3389/fendo.2018.00272)
Supplement: Supplementary file 1 [file data_sheet_1.docx]

***Supplemental data :***

New automatized method of 3D multiculture viability analysis based on confocal imagery: application to islets and mesenchymal stem cells co-encapsulation.

Clovis Chabert*^,a,1,2^, Camille Laporte^a,1^, Arnold Fertin^3^, Emily Tubbs^1^, Cécile Cottet-Rousselle^1^, Florence Rivera^4^, Magali Orhant-Prioux^5^, Anaick Moisan^5^, Eric Fontaine^1,6^, Pierre-Yves Benhamou^1,6^ and Sandrine Lablanche^1,6^

^a^ : Both authors contributed equally to this work.

^1^ Grenoble Alps University, Laboratory of Fundamental and Applied Bioenergetics (LBFA), and Environmental and System Biology (BEeSy), Grenoble, France; Inserm, U1055, Grenoble, France.

^2^  Laboratory « Adaptations au Climat Tropical, Exercice et Santé » (ACTES ;EA 3596), French West Indies University, Pointe-à- Pitre, Guadeloupe, France.

^3^ CNRS, TIMC-IMAG, University Grenoble Alpes, Grenoble, France

^4^ Microsyst. for Biol. & Health Dept., CEA-LETI, Grenoble, France.

^5^ Cell Therapy and Engineering Unit, EFS Auvergne Rhône Alpes, Saint Ismier, France.

^6^ Grenoble University Hospital, Grenoble, France.

Corresponding Author:

Dr Clovis CHABERT, Laboratoire de Bioénergétique Fondamentale et Appliquée, Inserm U1055, Université Grenoble Alpes, CS 40700, 38058 Grenoble cedex 09, France.

Tel. +590 89 31 72; Fax +33 476 514 218; email: clovis.chabert@gmail.com


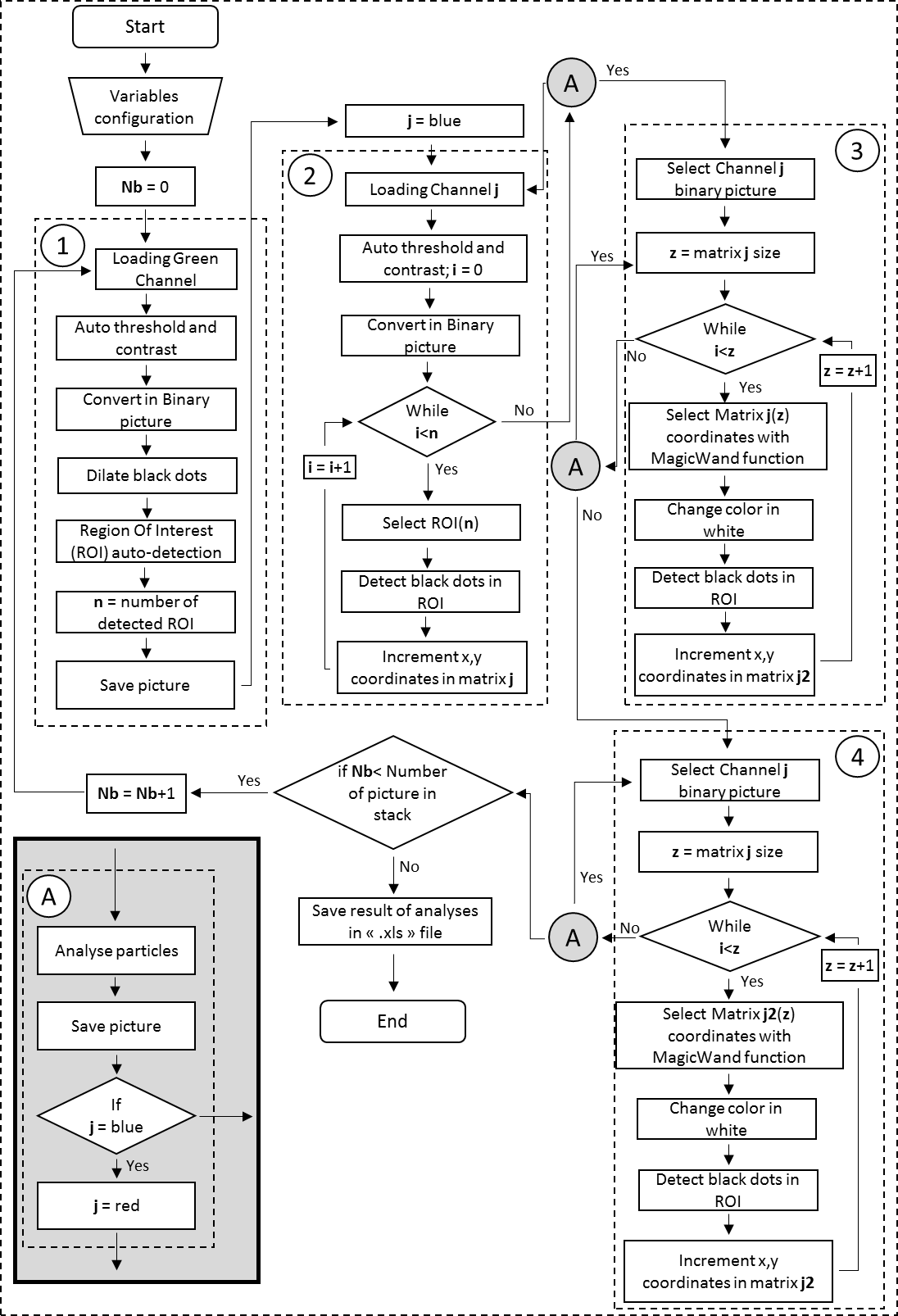


*Supplemental Figure 1: Algorithmic representation of the main steps of the software proceeding on the green channel ➀; the red and green channel ➁; the selection of the islets nuclei ➂ and the MSCs nuclei ➃. Green: Mesenchymal Stem Cells cytosol (Pkh); Blue: living nuclei (Hoechst stain). ROI: Region Of Interest.*
